# The Hippo pathway component *Wwc2* is a key regulator of embryonic development and angiogenesis in mice

**DOI:** 10.1038/s41419-021-03409-0

**Published:** 2021-01-22

**Authors:** Anke Hermann, Guangming Wu, Pavel I. Nedvetsky, Viktoria C. Brücher, Charlotte Egbring, Jakob Bonse, Verena Höffken, Dirk Oliver Wennmann, Matthias Marks, Michael P. Krahn, Hans Schöler, Peter Heiduschka, Hermann Pavenstädt, Joachim Kremerskothen

**Affiliations:** 1 Department of Nephrology, Hypertension and Rheumatology, University Hospital Münster, Münster, Germany; 2 Guangzhou Regenerative Medicine and Health Guangdong Laboratory, Guangzhou, P. R. China; 3 Department of Ophthalmology, University Hospital Münster, Münster, Germany; 4 Department of Neurobiological Research, University Aachen, Aachen, Germany; 5 Medical Faculty, University Münster, Münster, Germany

**Keywords:** Embryogenesis, Cells

## Abstract

The WW-and-C2-domain-containing (WWC) protein family is involved in the regulation of cell differentiation, cell proliferation, and organ growth control. As upstream components of the Hippo signaling pathway, WWC proteins activate the Large tumor suppressor (LATS) kinase that in turn phosphorylates Yes-associated protein (YAP) and its paralog Transcriptional coactivator-with-PDZ-binding motif (TAZ) preventing their nuclear import and transcriptional activity. Inhibition of WWC expression leads to downregulation of the Hippo pathway, increased expression of YAP/TAZ target genes and enhanced organ growth. In mice, a ubiquitous Wwc1 knockout (KO) induces a mild neurological phenotype with no impact on embryogenesis or organ growth. In contrast, we could show here that ubiquitous deletion of Wwc2 in mice leads to early embryonic lethality. Wwc2 KO embryos display growth retardation, a disturbed placenta development, impaired vascularization, and finally embryonic death. A whole-transcriptome analysis of embryos lacking Wwc2 revealed a massive deregulation of gene expression with impact on cell fate determination, cell metabolism, and angiogenesis. Consequently, a perinatal, endothelial-specific Wwc2 KO in mice led to disturbed vessel formation and vascular hypersprouting in the retina. In summary, our data elucidate a novel role for Wwc2 as a key regulator in early embryonic development and sprouting angiogenesis in mice.

## Introduction

Mammalian embryogenesis is a highly regulated process that includes specification of cell fate, targeted cell migration, and controlled cell proliferation. Altered expression or mutations of crucial genes can lead to a disturbed cell differentiation, deregulated organ development, impaired angiogenesis, and finally embryonic lethality.

Determination of cell fate starts very early in embryonic development. Mouse embryos at the blastocyst stage consist of two different cell types, (i) the inner cell mass (ICM) that later forms the embryo as well as extraembryonic structures (e. g. the yolk sac) and (ii) an surrounding layer of trophectodermal cells (TE) that will give rise to the embryonic portion of the placenta^1,2^. The Hippo signaling pathway and its key components, the co-transcription factor Yes-associated protein (YAP) and its paralog Transcriptional coactivator-with-PDZ-binding-motif (TAZ), are crucial for early embryogenesis as they determine cell-type-specific gene expression in the TE and the ICM^3–5^. In the TE cells, the Hippo pathway is silent which results in an enhanced activity of nuclear YAP/TAZ. In contrast, in cells of the ICM, Hippo pathway activity is high preventing YAP/TAZ-mediated gene transcription^6^.

Besides its role in cell fate determination during embryogenesis, the highly conserved Hippo signaling pathway is known to regulate postnatal organ growth in all organisms from fly to man^7–10^. In mammals, impaired Hippo signaling results in nuclear import of YAP/TAZ, inhibition of apoptosis, and enhanced cell proliferation. Nuclear YAP activity depends on its phosphorylation status, which prevents transport of YAP/TAZ into the nucleus and marks these proteins for proteasomal degradation^8,10^. These events are controlled by a growing number of upstream effectors and a cytosolic core kinase cascade culminating in YAP/TAZ phosphorylation by the Large tumor suppressor (LATS) kinases 1 and 2^11,12^. The family of WWC proteins, composed of WWC1 (also called kidney-and-brain (KIBRA) protein), WWC2 and WWC3, have a crucial impact on Hippo signaling because they enhance LATS1/2 kinase activity and subsequently YAP/TAZ phosphorylation^13,14^.

The structural similarity and a common subset of interacting partners indicate a redundant function of the WWC proteins^14,15^. Consequently, a ubiquitous knockout (KO) of *Wwc1* expression in mice (that have lost the *WWC3* gene during evolution) leads to an only mild neurological phenotype but has no crucial impact on embryogenesis or organ development likely due to *Wwc2*-mediated compensation^16,17^. In contrast, a hepatocyte-specific double KO of both *Wwc1* and *Wwc2* in mice results in Hippo pathway-dependent organ overgrowth and subsequent liver carcinoma^14^.

Recent data have shown that murine *Wwc2* is already expressed in the blastocyst, whereas *Wwc1* expression is almost undetectable at this stage^18^. To investigate a putative *Wwc2*-specific function in early embryogenesis, we generated a ubiquitous deletion of *Wwc2* in mice and observed embryonic lethality around day E11.5. Analysis of *Wwc2* KO embryos revealed growth retardation effects, an impaired embryonic vessel formation and a disturbed vascularization within the placenta. A whole-transcriptome analysis of *Wwc2* KO embryos at E11.5 demonstrated a strong deregulation of gene expression with an impact on Hippo signaling, cell metabolism, and vascular development. Furthermore, an inducible and endothelial-specific *Wwc2* KO in perinatal mice led to impaired vessel formation and vascular hypersprouting in the retina indicating a crucial role for *Wwc2* in angiogenesis.

## Results

### **E**mbryonic lethality in *Wwc2* KO mouse embryos

To analyze *Wwc2* function during embryonal development, a transgenic mouse line with a ubiquitous knockout of *Wwc2* was generated. For this, animals from a floxed *Wwc2* (*Wwc2*^fl/fl^) line^14^ were crossed with mice from a phosphoglycerate kinase (*Pgk*) 1 driven *Cre* recombinase deleter line (*Pgk-Cre*), in which target genes can be ubiquitously inactivated from early embryonic stages on^19^. The *Wwc2* genotype of the individual offspring (4 weeks old) was verified via PCR analysis (Supplemental Fig. S1A). From a total number of 83 analyzed mice, no animal with a homozygous *Wwc2* KO *(Pgk-Cre-Wwc2*^KO/KO^) could be found, but 28 wild-type (*Wwc2*^+/+^) and 55 heterozygous animals (*Pgk-Cre-Wwc2*^KO/+^). The last-named were vital and phenotypically normal just as *Pgk-Cre*-negative *Wwc2*^fl/fl^ mice. These findings pointed to intrauterine death of embryos lacking *Wwc2* expression. To determine the onset of embryonic lethality in more detail, we next isolated embryos at different developmental stages from embryonic day (E) 6.5 to E14.5 and determined their genotype. For this, *Wwc2*^fl/fl^ animals were crossed with mice from a zona pellucida glycoprotein 3 (*Zp3*) *Cre* recombinase deleter line (*Zp3*-Cre), in which target genes can be inactivated from growing oocytes in a *Zp3* promoter-dependent manner^20^. Normal Mendelian frequencies of wild-type (*Wwc2*^+/+^), heterozygous (*Zp3-Cre-Wwc2*^KO/+^) and homozygous (*Zp3-Cre-Wwc2*^KO/KO^) embryos were observed until E11.5 (Supplemental Tab. 3). Whereas *Wwc2*^*+/+*^ and heterozygous *Zp3-Cre-Wwc2*^KO/+^ embryos showed normal development after E11.5, homozygous *Zp3-Cre-Wwc2*^KO/KO^ embryos (called *Wwc2* KO later on) were degenerated or/and resorbed (Supplementary Fig. S1B). A Western blot analysis confirmed the lack of WWC2 protein in the E11.5 *Wwc2* KO embryos (Supplementary Fig. S1C).

**Fig. 1:**
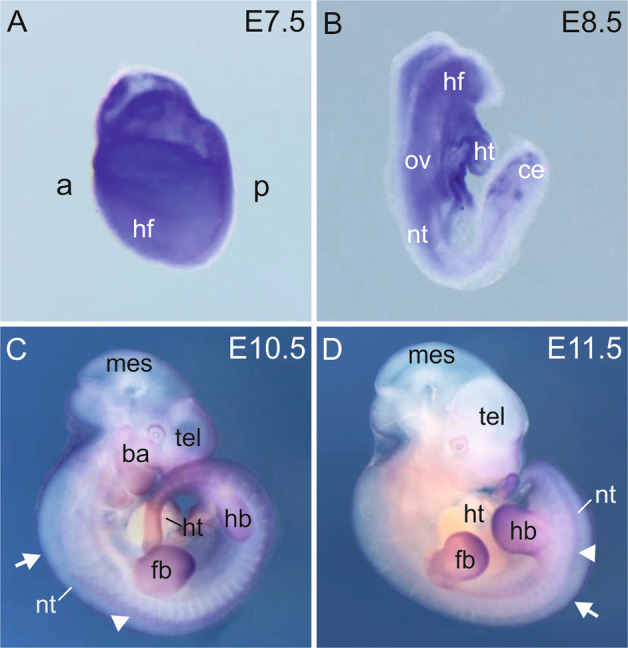
*Wwc2* expression in mouse embryos from E7.5 to E11.5. Spatial expression of *Wwc2* mRNA in E7.5 (**A**), E8.5 (**B**), E10.5 (**C**), and E11.5 (**D**) mouse embryos. White arrowheads mark expression in dorsal root ganglia, white arrows show expression in the roof plate of the neural tube. From E8.5 onwards extraembryonic membranes were removed. a anterior, ba branchial arches, ce caudal end, fb forelimb bud, hf head fold, ht heart anlage, hb hindlimb bud, nt neural tube, mes mesencephalon, ov otic vesicle, p posterior, tel telencephalon.

To gain more insight into the putative function of *Wwc2* in embryogenesis, we investigated the spatial and temporal expression of *Wwc2* during normal embryonic development. As immunohistochemical studies were not possible for WWC2 due to the lack of suitable antibodies, we used whole-mount in situ hybridization (WISH) to detect *Wwc2* mRNA in wild-type mouse embryos between E7.5 and E11.5. *Wwc2* transcripts were ubiquitously detectable at E7.5, in both embryonic and extraembryonic tissues (Fig. 1A). At E8.5, *Wwc2* expression was mainly visible in mesenchymal tissues (Fig. 1B). In E10.5 and E11.5 embryos, *Wwc2* mRNA was detectable in the surface ectoderm, with domains of enrichment in the branchial arches and the posterior-distal region of the limb anlagen (Fig. 1C, D). In addition to superficial ectodermal expression, *Wwc2* expression was found in the roof plate of the neural tube and the dorsal root ganglia (Fig. 1C, D). To get a more quantitative analysis of *Wwc2* mRNA expression during embryogenesis, we isolated whole mRNA from embryos at different stages and performed quantitative reverse transcriptase polymerase chain reaction (qRT-PCR). This approach revealed that *Wwc2* expression is already present at E8.5 and that the expression level remains stable until E13.5 with only minor changes (Supplementary Fig. S2).

### Placenta defects in *Wwc2* KO embryos

**Fig. 2:**
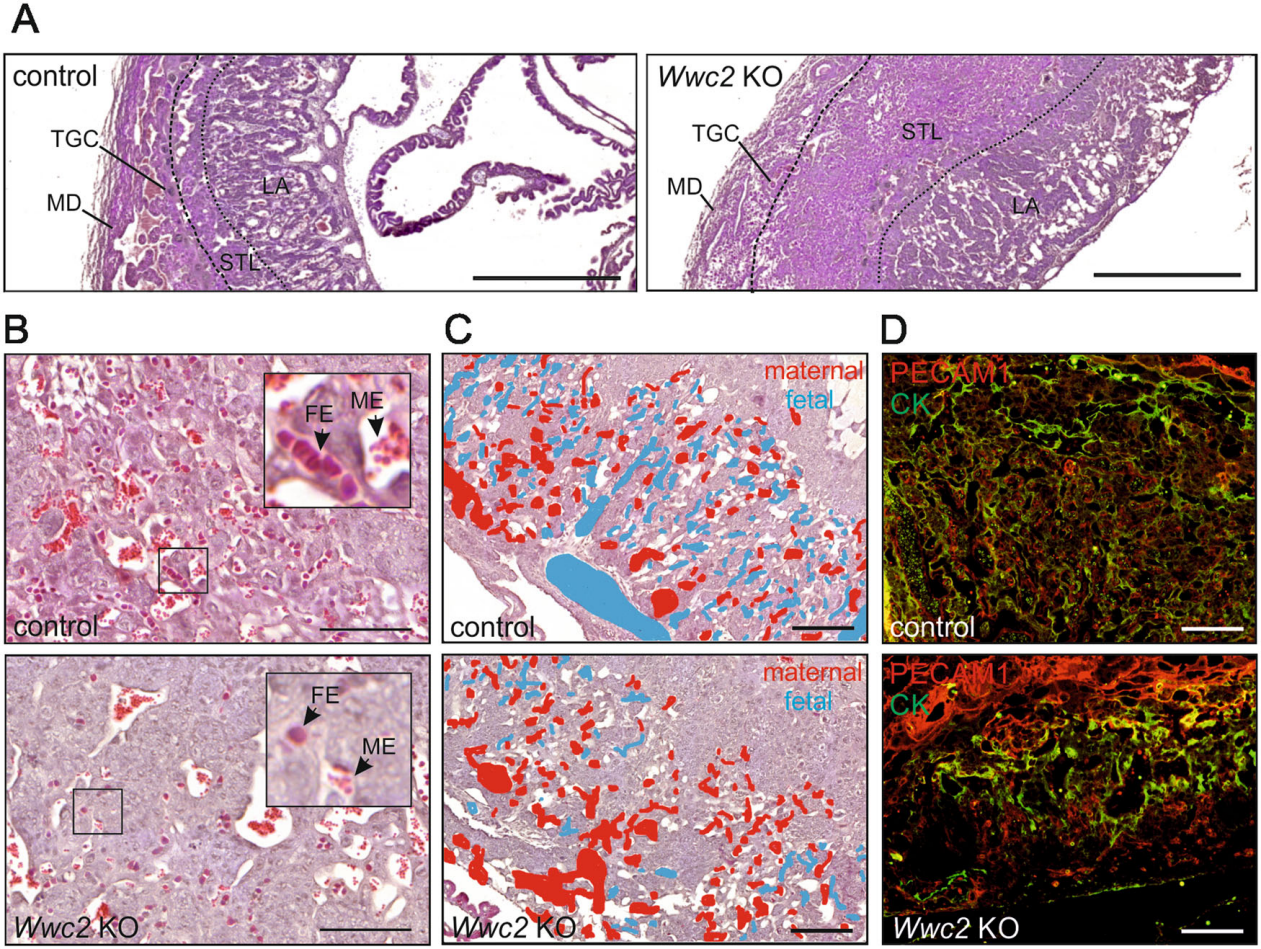
Defects in placenta organization and impaired labyrinth vascularization in *Wwc2* KO embryos. **A** PAS staining of control and *Wwc2* KO placenta at E11.5 indicate a disturbed and unorganized layer formation. MD maternal decidua, TGC trophoblast giant cell, STL spongiotrophoblast layer, LA labyrinth. Dashed lines mark areas of the different tissue layers. Scale bar = 1 mm. **B** H&E staining of placenta sections were used for the identification of maternal arterial sinuses and embryonic blood vessels. Insets show a magnification with small, red, non-nucleated maternal erythrocytes (ME) and bigger, purple, nucleated fetal erythrocytes (FE). Scale bar = 100 µm. **C** Labeling of fetal (blue) and maternal (red) erythrocytes indicated a disturbed organization and impaired permeation of fetal blood vessels in the labyrinth system. Scale bars = 200 µm. **D** Immunofluorescence staining of sections from control and *Wwc2* KO placenta using antibodies against PECAM1 (red; endothelial cell marker) and pan-cytokeratin (CK, green; trophoblast marker) revealed reduced vascularization of the labyrinth. Scale bars = 200 µm.

The WISH results provide novel insight into the developmental regulation of *Wwc2* expression. However, the data did not explain the reason(s) for the embryonic lethality observed in *Wwc2* KO mice. As embryonic death around E11.5 in mice is typically linked to placental defects and a disturbed nutrition of the embryo^21,22^, we next analyzed the structure of the placenta from control *(Wwc2*^+/+^ or *Zp3-Cre*-*Wwc2*^KO/+^) and *Wwc2* KO embryos, respectively. Murine placenta is composed of the maternal decidua (MD), a single layer of trophoblast giant cells (TGC), a layer of embryonic spongiotrophoblasts (STL) and the labyrinth system (LA) that ensures the exchange of nutrients and oxygen between maternal and fetal vessels^23–26^. PAS staining of placenta sections from embryos at E11.5 revealed an unorganized structure of the anatomical layers in *Wwc2* KO embryos. The TG layer, normally arranged in a chain-like structure separating the MD and the STL, was dispersed and hardly visible (Fig. 2A). In addition, the STL was thickened in the *Wwc2* KO placenta compared to the control, whereas the LA appeared more compact (Fig. 2A).

Vascularization within the labyrinth system is a highly regulated process ensuring the metabolic requirements of the embryo with a supply of essential components from maternal blood^24,26–28^. Maternal and fetal blood vessels in the labyrinth system are distinguishable by the presence of different types of erythrocytes. Maternal erythrocytes (ME) appear as small, non-nucleated cells, whereas fetal erythrocytes (FE) are bigger and nucleated^29–31^. Hematoxylin and eosin (H&E) staining of placenta sections from E11.5 *Wwc2* KO embryos revealed a markedly reduced number of fetal blood vessels and FE in the LA layer (Fig. 2B, C). Furthermore, an immunofluorescence staining of the placental vasculature using antibodies directed against the endothelial marker Platelet/endothelial cell adhesion molecule-1 (PECAM1) and the trophoblast cell marker cytokeratin (CK) indicated a failure of embryonic vessels to permeate into the STL of the *Wwc2* KO placenta (Fig. 2D).

### *Wwc2* deficiency leads to an impaired angiogenesis in the yolk sac and embryo proper

The observed defects in the placental structure *of Wwc2* KO embryos are likely to cause an insufficient supply of the embryo with nutrients and oxygen that finally leads to embryonic death around E11.5. To test whether *Wwc2* ablation leads to additional defects in the embryo itself, a tetraploid complementation assay was performed^32^. In this assay, extraembryonic tissue is formed by tetraploid cells, while the embryo is exclusively formed by diploid embryonic stem (ES) cells. Using wild-type tetraploid cells to ensure normal formation of extraembryonic tissue and ES cells from a line with a heterozygous *Wwc2* KO, several E19.5 embryos with a normal development were obtained in this assay. In contrast, no E19.5 embryo could be generated when ES cells with a homozygous *Wwc2* KO from three different lines were used (Supplementary Table 4). These data indicated extra-placental, embryo-specific defects in development that are linked to a *Wwc2* ablation.

**Fig. 3:**
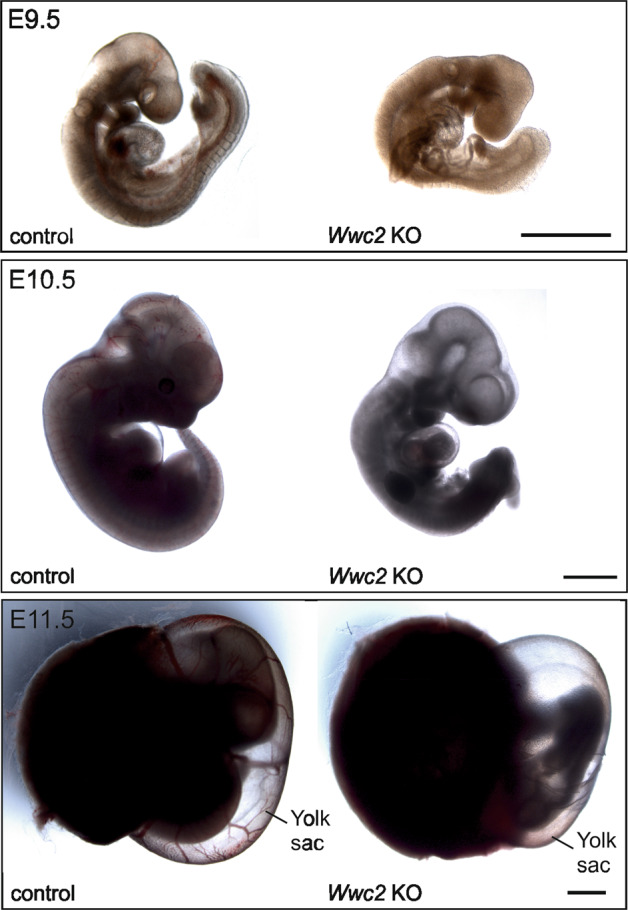
Growth retardation and disturbed blood flow in Wwc2 KO embryos and yolk sacs. *Wwc2* KO embryos at E9.5. or E10.5 appeared growth retarded and less developed when compared to control embryo. Furthermore, no blood-filled vessels are observed in *Wwc2* KO embryos (E10.5) and yolk sacs (E11.5). Scale bars represent 500 µm.

Next, we used *Wwc2*-deficient embryos between E9.5 and E11.5 for histological examination. Compared to controls, *Wwc2* KO embryos appeared growth retarded and less developed at E9.5, E10.5, and E11.5. Furthermore, in the absence of *Wwc2*, an almost complete loss of blood-filled vessels was observed in the embryos and the yolk sac at E11.5 (Fig. 3).

**Fig. 4:**
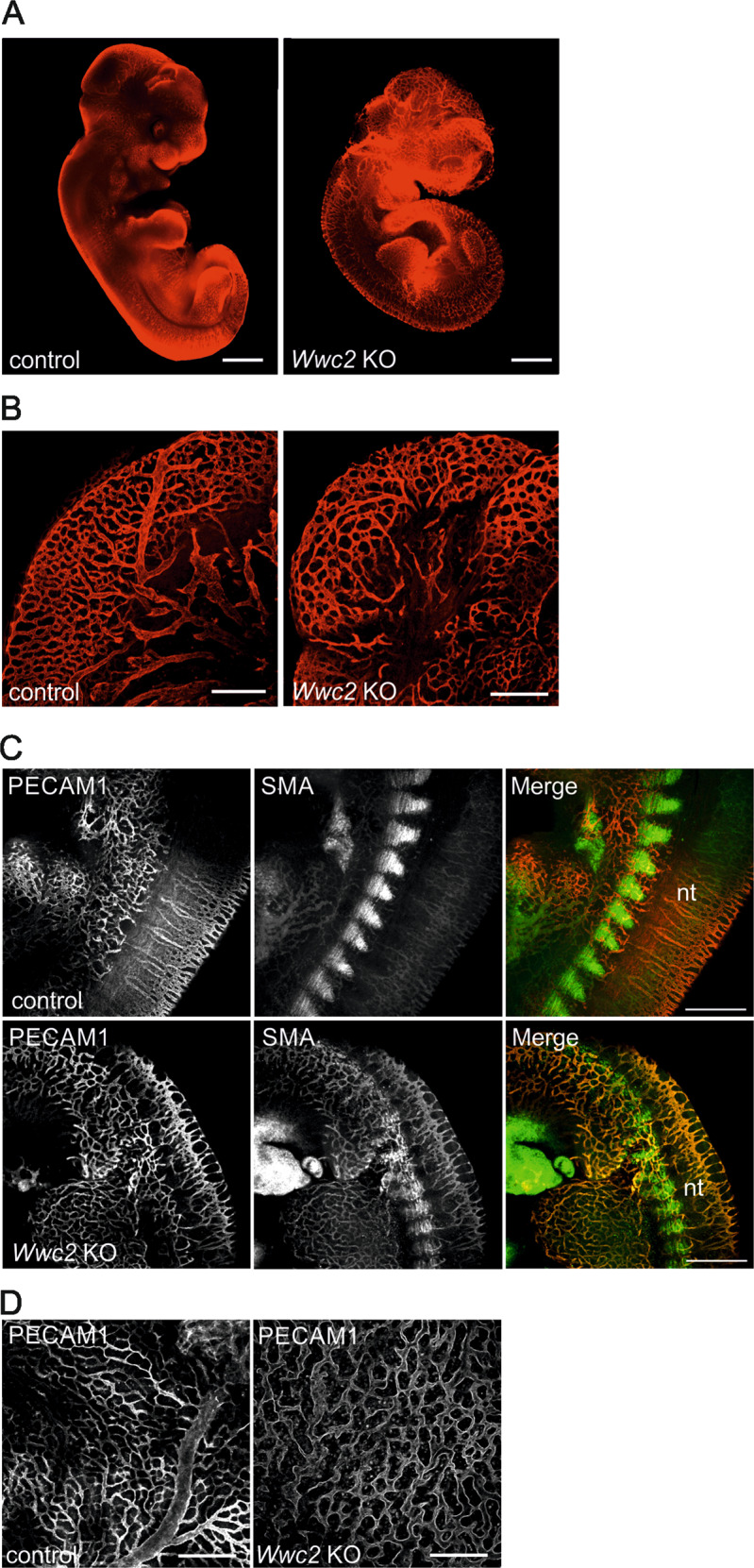
Impaired vascularization in Wwc2 KO embryo and yolk sac. **A** Whole mount staining of E11.5 embryos using antibodies against the endothelial marker PECAM1 revealed defects in the vascular system of the *Wwc2* KO embryo. Control embryo has normally organized and branched network of vessels. Scale bar = 500 µm. **B** Vascular patterning in the cranial region of E11.5 control and *Wwc2* KO embryos. Scale bars = 50 µm. **C** Whole mount staining of E11.5 embryos with PECAM1 and anti-SMA antibodies reveals a disorganized vascularization in the neural tube (nt) and an irregular pattern of pericytes in *Wwc2* KO embryos. Scale bar = 20 µm. **D** Whole mount staining of E11.5 yolk sacs with PECAM1 reveals a disturbed vessel branching in the *Wwc2* KO yolk sac. Scale bars = 20 µm.

The vascular structure of *Wwc2* KO embryos at E11.5 was further analyzed using whole mount immunostaining for PECAM1-positive, endothelial cells. This approach revealed that the vascular network was present but less organized in the absence of *Wwc2* (Fig. 4A). Cranial vessels in the *Wwc2* KO embryo showed an almost uniform diameter and resembled a honeycomb-like structure with no large vessels, which were present in the control embryos at this time point (Fig. 4B). A co-immunostaining of endothelial cells (PECAM1-positive) and pericytes, multifunctional mural cells wrapping around blood capillaries (positive for the marker protein smooth muscle actin (SMA)), revealed an irregular pattering of these cell types in the vascular system of *Wwc2* KO embryos (Fig. 4C). Furthermore, the architecture of the vascular system surrounding the neural tube (NT) was disturbed and the lateral branches between the intersomitic vessels were almost completely absent in the *Wwc2* KO embryo (Fig. 4C). Similar to the embryo trunk, the yolk sac of the *Wwc2* KO embryos lacked the typical hierarchical tree-like structure of the vascular network, instead vessels were organized in a more primitive honeycomb-like network (Fig. 4D).

### Whole-transcriptome analysis of *Wwc2* KO embryos

**Fig. 5:**
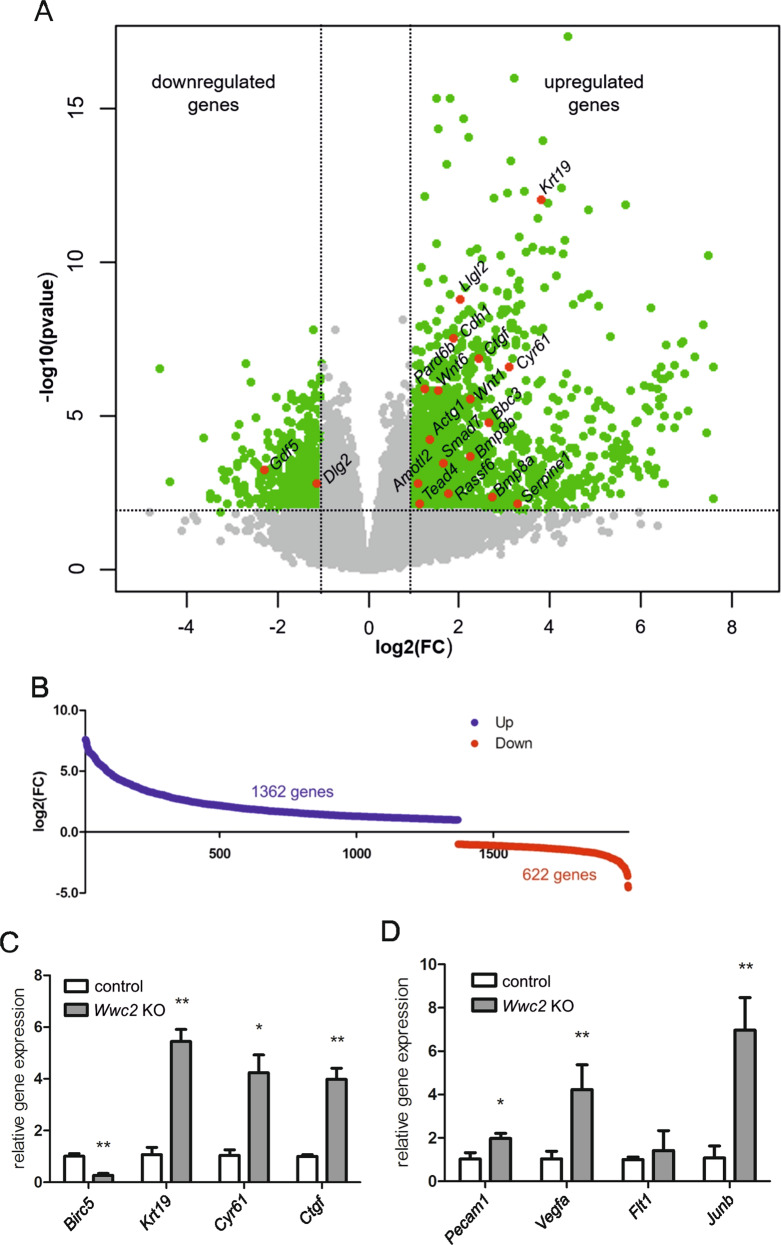
Differentially expressed genes in *Wwc2* KO embryos. **A** The whole transcriptome from wildtype (*n* = 3) and *Wwc2* KO (*n* = 4) embryos at E11.5 was compared. Volcano plot with (base 2) logarithmic fold change (FC) plotted against the negative (base 10) logarithmic *p*-value. Each of the 20,653 detected genes is represented by a dot. For an in-depth analysis, a threshold was set indicated by the dashed lines. Genes exceeding that threshold are marked in green, resulting in 1984 genes with an FDR-corrected *p*-value < 0.05 and a log2(FC) > 1 that were taken into account. Genes below that threshold are marked in gray and were ignored for further analysis. Genes related to the Hippo signaling are marked in red (KEGG pathway: Hippo signaling pathway). **B** Graph showing the group of 1984 significantly deregulated genes with FDR-corrected *p*-value < 0.05 and log2(FC) > 1 divided in upregulated genes (“Up”, 1362 genes, blue) and downregulated genes (“Down”, 622 genes, red). **C** qRT-PCR analyses with RNA isolated from control (*n* = 3) and *Wwc2* KO embryos (*n* = 3) at E11.5. The known YAP target genes *Krt19, Cyr61*, and *Ctgf* are significantly upregulated in *Wwc2*-deficient embryos. **D** Expression of the angiogenesis-related genes *Pecam1, Vegfa, Flt1*, and *JunB* is upregulated in *Wwc2* KO embryos.

To elucidate the function of *Wwc2* in embryonic development at the molecular level, a whole-transcriptome analysis by next generation sequencing was performed. In order to identify genes that are differentially expressed, whole RNA was isolated at E11.5 from wildtype (*Wwc2*^*+/+*^; *n* = 3), heterozygous (*Zp3-Cre-Wwc2*^KO/+^; *n* = 3), and homozygous (*Zp3-Cre-Wwc2*^KO/KO^; *n* = 4) embryos and the mRNA levels were determined. The FPKM (fragments per kilobase of transcript per million fragments mapped) value was used to normalize the number of reads to the mRNA length and the total read number in the measurement. With this normalization, mRNA levels can be compared in different samples taking both molar concentration and transcript length into account. The reads in the measurement allowed the identification of a total number of 20,653 genes. No significant differences within the transcriptome between wild type and heterozygous *Wwc2* KO embryos were detected (data not shown). In contrast, the transcriptomes obtained from the homozygous *Wwc2* KO embryos displayed remarkable changes when compared to the wildtype and heterozygous embryos (Fig. 5A). As expected, the group of differentially regulated genes (DEG) included a numerous known YAP/TAZ target genes that were upregulated in homozygous *Wwc2* KO embryos (Fig. 5A). For an in-depth analysis, a threshold was set in which only the differentially expressed genes with a log2 (fold change) greater than 1 were taken into account. As a result, a group of 1984 significantly differentially expressed genes could be identified including 1362 upregulated and 622 downregulated genes (Fig. 5B).

Unexpectedly, we observe that genes encoding proteins with a usually placenta-specific expression (e.g. members of the *prolactin family (Prl), pregnancy specific glycoproteins (Psg), trophoblast-specific proteins (Tpbp)*, placenta-specific cathepsin isoforms, and *carcinoembryonic antigen-related cell adhesion molecules* (*Ceacam)*^33,34^ were highly represented among the 30 most upregulated genes from *Wwc2* KO embryos (Supplementary Tab. 5). Other upregulated genes encoded proteins that are involved in DNA repair, protein degradation, and tissue remodeling. Among the 30 most downregulated genes, a remarkable number of genes have functions in the nervous system (Supplementary Tab. 6).

For further interpretation of the results, the annotation tool software from the Database for Annotation, Visualization, and Integrated Discovery (DAVID) was used to perform gene set enrichment analysis on Gene Ontology (GO) terms from the Uniprot “Tissue” or the “Biological Processes” data set to identify significantly enriched gene sets. The significantly upregulated genes could be significantly enriched in the three terms “Trophoblast stem cells”, “placenta” and “kidney” (Supplementary Fig. 3A). The significantly downregulated genes could be assigned to terms related to different brain regions (Supplementary Fig. 3A).

The gene set enrichment analysis for the ontology category “Biological Process” revealed that the upregulated genes in *Wwc2* KO embryos were enriched in the terms “angiogenesis“, “transcription and translation”, “metabolism”, “placenta development”, and “cell adhesion” (Supplementary Fig. 4A). The downregulated genes in *Wwc2* KO embryos were enriched in terms related to processes within the nervous system, e.g. “neuronal action potential” (Supplementary Fig. 4B).

*Gene set enrichment analysis* (GSEA), an alternative software tool for the analysis on differentially expressed genes, gave similar results when compared to the performed DAVID analysis (Supplemental Tables 7 and 8).

To confirm the transcriptome data indicating an effect of the *Wwc2* KO on genes involved in Hippo signaling and angiogenesis, we used qRT-PCR analysis with RNA isolated from E11.5 control and homozygous *Wwc2* KO embryos. These experiments revealed an enhanced expression of the YAP target genes *Krt19, Cyr61*, and *Ctgf* in *Wwc2* KO embryos that likely resulted from a decreased Hippo signaling (Fig. 5C). Furthermore, a significantly upregulation of the angiogenesis-related genes *Pecam1, Vegfa*, and *Junb* in the *Wwc2* KO embryos was detected probably causing an imbalance in angiogenesis (Fig. 5C).

### Postnatal angiogenesis in the retina of mice with an endothelial-specific *Wwc2* KO

The data from the phenotypic analyses clearly revealed an impact of the ubiquitous *Wwc2* KO on angiogenesis inducing an early embryonic lethality. To gain more insight into the endothelial-specific function of *Wwc2* in postnatal angiogenesis, we next examined the growth of retinal vessels in the absence of *Wwc2* expression. For this approach, we crossed *Wwc2*^fl/fl^ mice with mice from a Cadherin 5 (Cdh5)-*Cre*ER^T2^ line in which endothelilal-specific activity of Cre recombinase is induced by Tamoxifen treatment^35^. To exclude a putative compensatory effect of *Wwc1* in the absence of *Wwc2* in endothelial cells, we included mice from a *Wwc1*^fl/fl^ -*Wwc2*^fl/fl^ line^14^ in these experiments.

**Fig. 6:**
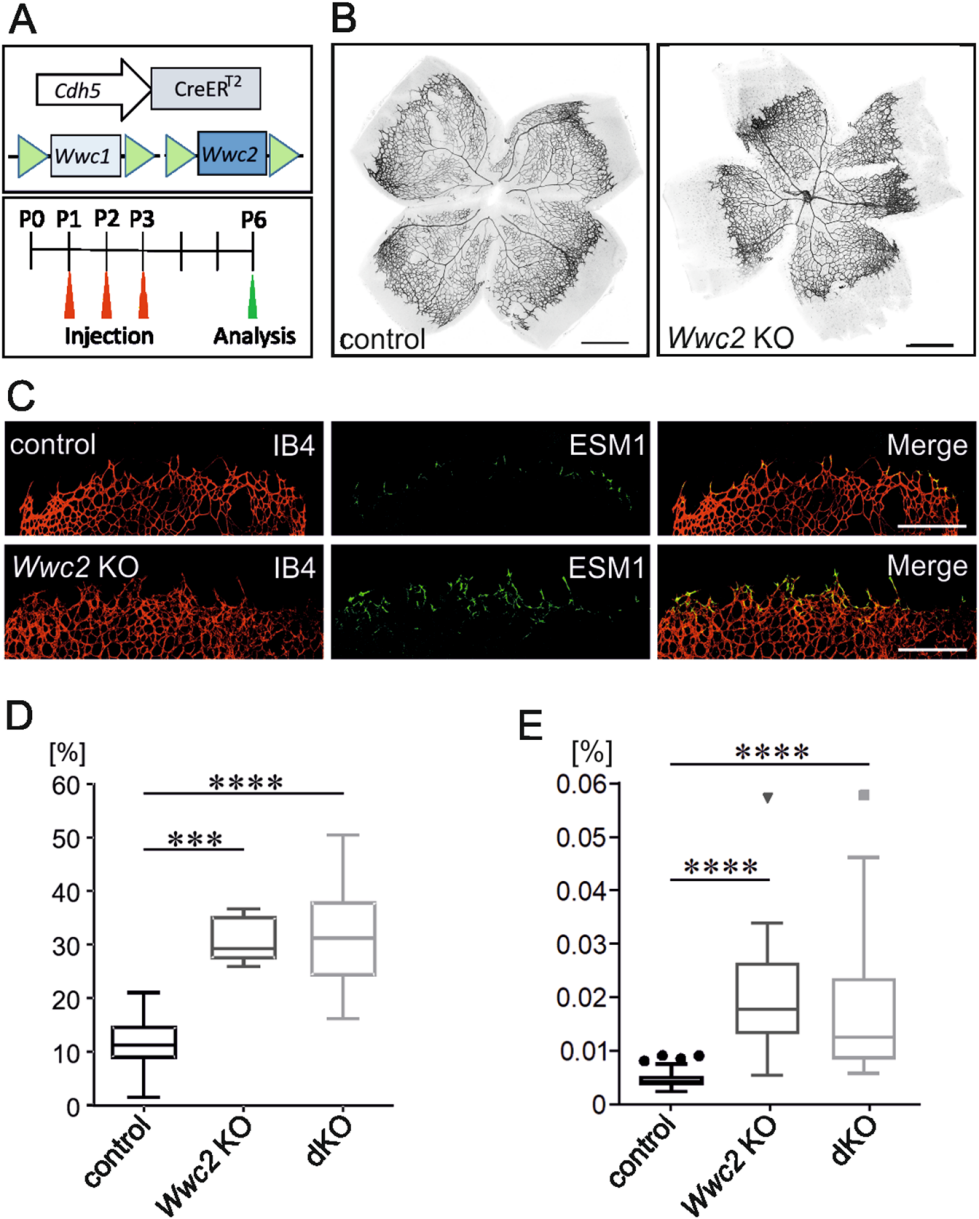
Disturbed postnatal angiogenesis and vascular hypersprouting in retina from *Wwc2* KO mice. **A** Scheme describing the endothelial-specific deletion of *Wwc1* and *Wwc2* in postnatal *Cdh5*-*Cre*ER^T2^ mice that was achieved by an intragastric Tamoxifen injection (50 µl of a Tamoxifen stem solution (1 mg/ml)) at P1–P3. At P6, mice were euthanized and retinas were dissected. **B** IB4-stained vessels in retina flat mounts from control and *Wwc2* KO mice. **C** Vascular front region in flat mounts stained for vessels (IB4) and leading tips cells (ESM1). **D** Percentage of the area of vascular hypersprouting compared to total vascular area in controls and *Wwc2* KO retinas. **E** Ratio of the number of vascular endpoints in relation to the total vessel length in controls and *Wwc2* KO retinas. Data in **D** and **E** are presented as box plots according to Tukey, i.e. whiskers have a maximum length of 1.5 interquartile length, and the symbols (circles, triangle, square) represent outliers. Statistical significance of differences is indicated as follows: ***p < 0.001, ****p < 0.0001.

Postnatally, *Cre*-mediated gene deletion was achieved through a triple intragastric Tamoxifen injection at P1–P3. *Cre*-negative animals were used as controls. At P6, mice were euthanized, and retinas were dissected for further analysis (Fig. 6A). Staining of retina flat mounts with Isolectin B4 (IB4) revealed a highly differentiated vessel network in control and *Wwc2* KO retina. However, retinas from *Wwc2* KO mice displayed an impaired vessel growth with hypersprouting and increased vessel density at the vascular front (Fig. 6B).

Postnatal sprouting of vessels is controlled by a highly complex interplay between guiding tip cells and stalk cells^36,37^. Pathologic hypersprouting is frequently connected to an increased number of tip cells^38–40^. Indeed, staining of retina with IB4 and antibodies against the tip cell marker *Endothelial cell-specific molecule* (Esm1)^41^ demonstrated an increased number of tip cells in the vascular front region in *Wwc2* KO mice (Fig. 6C). A quantitative analysis of the observed retina phenotype revealed that the percentage of the vascular hypersprouting region in relation to the overall vascularized area was significantly higher in *Wwc2* KO mice when compared to controls. Double KO (dKO) of both *Wwc1* and *Wwc2* did not further enhance this effect (Fig. 6D). Another evidence of vascular hypersprouting in *Wwc2* KO retinas was the significantly enlarged number of vessel endpoints in relation to the total vessel length in the analyzed images (Fig. 6E). Again, there was no difference between WWC2 KO and double KO animals.

## Discussion

The Hippo signaling pathway is involved in the regulation of cell proliferation, apoptosis and organ growth. Besides the core kinase cascade, Hippo signaling is controlled through an increasing number of upstream regulators including the WWC proteins^10–12^. The WWC protein family was identified to induce LATS1/2 activity that promotes cytoplasmic retention of YAP in vitro^13^. Using KO mouse models*, Wwc1* and *Wwc2* were shown to have a Hippo pathway-dependent impact on postnatal organ growth and tumorigenesis. A hepatocyte-specific KO of *Wwc1* and *Wwc2*, but not of a single WWC protein, leads to liver overgrowth and subsequent carcinoma formation in mice^14^. These findings confirm the important role of WWC proteins for Hippo signaling in vivo and furthermore suggest a redundant function of the different WWC family members.

Besides its role in control of postnatal cell proliferation, Hippo signaling is required for processes during embryogenesis. Consequently, gene KO in mice affecting the Hippo pathway frequently result in embryonic lethality and resorption that mainly occur between E8.5 and E13.5^42–44^. Mouse embryos harboring a deletion of *Ww45* or *Mst1/2* display vasculature defects and growth retardation as well as a disturbed placental development^45,46^. Our report confirms the crucial role of the WWC gene family for Hippo signaling but further elucidate the specific role of *Wwc2* in early embryogenesis.

A *Wwc2* KO in mice leads embryonic lethality around day E11.5. Until this stage, embryonic morphogenesis, embryo segmentation, and organogenesis were present but in an impaired manner (Fig. 3). We speculate that *Wwc2* deficiency is probably tolerated within very early steps of embryogenesis but become crucial between E8.5 and E11.5. During this embryonic period, the development of the murine placenta takes place that ensures the supply of the fetus with nutrients and oxygen from the mother^26,28^. Indeed, *Wwc2* KO embryos showed a disturbed organization of placenta structures with impaired vessel formation and vascularization (Fig. 2). These placental defects seem to be an important, but not the exclusive reason for the observed embryonic lethality. Additionally, vascular development in the embryo proper and extraembryonic tissues was impaired. In control E11.5 embryos, cranial vasculature and the vessels in the yolk sac remodeled from a honeycomb-like structure to hierarchical tree-like structures with large and small blood vessels. However, vasculature in *Wwc2* KO embryos was still honeycomb-like with all vessels of similar size indicating failure of remodeling. Since embryo and extraembryonic tissues are highly connected and interdependent, the impaired vascularization within the *Wwc2* KO embryo might lead to disturbed blood circulation and (indirectly) to placental failure. Another scenario is based on defects in the placental vasculature that leads to malnutrition of the embryo, growth retardation and finally embryonic death. However, results of the tetraploid complementation assay indicate that even in conditions, when placental development was normal, KO of *Wwc2* in the embryo proper led to the death of the embryo.

YAP was shown to be essential for the formation of the vascular system in mice^40,47–49^. The vascular endothelial growth factor (VEGF) induces nuclear import and activation of YAP that results in enhanced transcription of the YAP target genes *Ctgf* and *Cyr61* activating angiogenesis^48,50^. Indeed, we demonstrated that deletion of *Wwc2* leads to increased levels of *Cyr61* and *Ctgf*, but also forces the expression of angiogenesis-related genes including *Vegfa, Pecam1* and *JunB* (Fig. 5C).

Recent studies revealed that vessel remodeling and vascular sprouting are sensitive to a deregulation of the Hippo pathway and YAP-dependent gene expression^47,49^. During the normal development of the murine vascular system, a honeycomb-like network of endothelial-lined, polygonal channels within the embryo and the yolk sac, and remodels to form a branched network of large and small vessels surrounded by smooth muscle cells. This process is described to be highly dependent on the expression of a variety of genes that are related to the Hippo pathway^40,51^. In the *Wwc2*-deficient embryos and yolk sacs, vessels are arranged in the described honeycomb-like network without any hierarchical architecture indicating a developmental arrest and a failure of vessel remodeling (Fig. 4). The altered distribution of vascular endothelial cells and pericytes surrounding the blood vessels strengthen these observations (Fig. 4) although *Wwc2* seems to be dispensable for the differentiation of endothelial cells or pericytes from their precursor cells itself.

Unexpectedly, our transcriptome study revealed a strongly upregulated expression of placenta-specific genes in the *Wwc2* KO embryo pointing to deregulation of cell fate determination at the preimplantation stage. In the blastocyst, proper differentiation of ICM and TE cell lineages depend on the expression of the transcriptional factor *Cdx2* that in turn requires nuclear YAP activity^2–5^. YAP is predominantly cytosolic in cells of the ICM, whereas in the TE cells, YAP displays a nuclear enrichment and induces the expression of genes associated with trophoblast specification including Cdx2^52^. Consequently, activity of the core kinase cascade within the Hippo pathway is low in TE cells but high in the ICM preventing nuclear YAP accumulation. In *Wwc2* KO embryos, we observed an upregulation of *Cdx2* and *Gata3* expression that points to a deregulation of the gene profile in ICM cells likely due to low LATS1/2 activity and accumulation of nuclear YAP at the blastocyst stage.

It is worth to note, that YAP activity at the blastocyst stage is also regulated by the Angiomotin (AMOT) family consisting of AMOT, Angiomotin-like protein 1 (AMOTL1) and Angiomotin-like protein 2 (AMOTL2)^53–55^. Angiomotins anchor YAP at the cytoskeleton preventing its nuclear import and transcriptional activity^56^. WWC2 was recently shown to prevent Angiomotins from proteasomal degradation^14^. Therefore, we speculate that loss of WWC2 expression does not only lead to decreased LATS1/2 activity but to lower Angiomotin levels both enhancing nuclear YAP activity in ICM cells.

A ubiquitous *Wwc2* KO led to various defects in development including a disturbed cell fate determination at the blastocyst state and deregulated vessel formation in the later steps of embryogenesis. In addition, our transcriptome approach and the gene set enrichment analysis of downregulated genes revealed an enrichment of GO terms related to the nervous system and brain development in *Wwc2* KO embryos (Supplementary Figs. 3 and 4). Upregulated genes were enriched in GO terms related to glycolysis, hypoxia, regulation of gene expression, and protein translation in *Wwc2* KO embryos (Supplementary Fig. 3). Most of these deregulated gene expression patterns might represent secondary effects caused by the disturbed angiogenesis and deficiencies in embryo nutrition. As a consequence, several survival mechanisms might be activated to compensate cell and tissue death and to prevent degeneration.

We propose that the impaired angiogenesis in *Wwc2* KO embryos is likely caused by an inhibited Hippo pathway and an enhanced YAP/TAZ transcriptional activity in endothelial cells. This hypothesis is supported by our studies on postnatal vessel formation in the retina from mice with an endothelial-specific *Wwc2* KO that demonstrated an increased proliferation of tip cells and vascular hypersprouting (Fig. 6B, C). Tip cells are located at the ends of angiogenic sprouts and their filopodial protrusions directed vessel growth toward angiogenic attractants^57–59^. Recent data showed that *Cyr61*, a known YAP/TAZ target gene, stimulates tip cell proliferation and activity^60^. Therefore, loss of endothelial *Wwc2* expression might activate YAP/TAZ-dependent *Cyr61* expression that in turn leads to enhanced tip cell activity and disturbed vessel sprouting.

In summary, the results presented here elucidate the function of *Wwc2* in embryogenesis and sprouting angiogenesis in mice and shed more light onto the role of the Hippo pathway on these processes. During the preparation of this manuscript, Virnicchi et al.^61^ presented data on the crucial role of *Wwc2* on cell proliferation during preimplantation of mouse embryos. Although their results partially differ from our data (likely due to the different experimental approach), this study further underlines the crucial role of *Wwc2* in mouse embryogenesis.

## Materials and methods

### Mice

Animal experiments were performed in accordance with national guidelines and were approved by the local authorities of North Rhine-Westphalia, Germany.

Generation of mice with a floxed *Wwc2* gene (*Wwc2*^fl/fl^) and mice harboring floxed *Wwc2* and *Wwc1* genes (*Wwc1*^fl/fl^-*Wwc2*^fl/fl^) were described before^14^. *Wwc2*^fl/fl^ mice were crossed with Phosphoglycerate kinase (*Pgk)* promotor-driven *Cre* deleter mice to obtain ubiquitous *Wwc2* deletion^19^. Crossing *Wwc2*^fl/fl^ mice with mice from a Zona occludens protein 3 (*Zp3*) promotor-driven *Cre* deleter line was used to remove both maternal and zygotic *Wwc2* expression^20^.

The Cadherin 5 (*Cdh5*)-*Cre*ER^T2^ mouse line, enabling an inducible endothel-specific activation of *Cre* expression, was described earlier^35^ and was a generous gift from R. Adams (Max-Planck-Institute for Molecular Biomedicine, Muenster, Germany). To generate a postnatal endothelial-specific knockout, mice were injected on postnatal day 1 (P1), P2, and P3 intragastric with 50 µl Tamoxifen (Sigma-Aldrich) at a concentration of 1 mg/mL in corn oil (Sigma-Aldrich).

### Genotyping

Genotyping was performed as described earlier^14^. In brief, ear clips or embryonic tissues were lysed in DirectPCR-Tail Lysis buffer (Peqlab/VWR, Germany) containing Proteinase K (Qiagen) for 12 h at 56 °C. Proteinase K was heat inactivated for 5 min at 95 °C. Lysates were centrifuged at 14,000 × *g* for 10 min and the supernatants were used for genotyping by PCR analysis. The used primers are listed in Supplementary Tab. 1.

### Generation of ES cell lines and tetraploid complementation assay

To establish embryonic stem (ES) cell lines, E3.5 blastocysts were flushed from uterus of *Zp3-Cre-*Wwc2^fl/fl^ female mice mated with *Wwc2*^KO/+^ male mice and cultured on gamma-ray inactivated mouse embryonic fibroblasts with a standard procedure^57^ after removal of the zonae pellucidae by acidic Tyrode’s solution (Sigma-Aldrich). From 37 blastocysts collected, 20 ES lines were established. Seven of ES cell lines showed the homozygous *Wwc2* ablation.

For the tetraploid complementation assay, two-cell embryos were flushed 42 h post-hCG from oviducts of mice with M2 medium and fused with a Cellfusion CF-150/B apparatus in a 250-μm gap electrode chamber (BLS Ltd., Budapest, Hungary) containing 0.3 M Mannitol with 0.1 mM MgSO_4_, 0.1 mM CaCl_2_, and 0.3% bovine serum albumin (Sigma-Aldrich). An initial electrical field of 2 V was applied to the embryos, followed by one peak pulse of 50 V for 35 μs. Embryos were washed and immediately transferred back into potassium simplex optimized medium (KSOM) medium^62^ in a 37 °C incubator. Fused tetraploid embryos were cultured overnight to four-cell stage and used for aggregation as reported with a slight modification^63^. Briefly, clumps of trypsin-treated ESCs (10–15 cells) were transferred into a dent in a drop of KSOM medium. Meanwhile, batches of 40–50 embryos were briefly incubated in acidified Tyrode’s solution until dissolution of their zonae pellucidae. Two embryos were placed in the dent next to cells to make one aggregate. All aggregates were assembled in this manner. After overnight culture at 37 °C in 5% CO_2_, blastocysts were transferred to each uterine horn of 2.5 days post coitum, pseudopregnant CD1 females that had been mated with vasectomized males. For cesarian derivation, recipient mothers were sacrificed at E19.5 and pups were quickly removed.

### Retina isolation

Isolation of retinas from postnatal mice and preparation of flat mounts were performed according to a published protocol^64^.

### Immunohistochemistry and immunofluorescence staining

Mouse embryos or placenta tissues were fixed in paraformaldehyde (PFA) for 4–24 h, washed with PBS, and embedded in Paraffin following standard procedures. Sections (4 µm) were deparaffinized in Tissue-Clear (Sakura Finetek, USA) for 20 min, and rehydrated by passage through a decreasing alcohol gradient into distilled water. The sections were stained with hematoxylin and eosin (H&E) or periodic acid Schiff (PAS) stain following standard staining protocols. The primary antibodies used are listed in Supplementary Tab. 2. Antibodies were diluted in 1% bovine serum albumin (BSA) in PBS and incubated overnight. As secondary antibody the Vectashield ABC system (Vector Laboratories) was used with DAB (Peroxidase Substrate Kit, Vector Laboratories) as a substrate.

For immunofluorescence staining, embryonic tissues, or placenta were embedded in O.C.T. (Sakura Finetek) and immediately frozen in a bath of super-cooled 2-methylbutane. The sections (7 µm) were fixed with PFA, pretreated with 0.2% Triton X-100 and blocked with 5% BSA in PBS for 1 h. Primary antibodies are listed in Table 2 of the supporting information. Antibodies were diluted in 1% BSA in PBS, applied to sections and incubated overnight at room temperature. To visualize antigens by immunofluorescence staining, the sections were washed with PBS and incubated with secondary antibodies coupled to fluorochromes (obtained from Invitrogen). Sections were analyzed using a fluorescence microscope (Zeiss, Observer Z1, HXP120, Axiocam MRm) and AxioVision 4.8 (Zeiss).

The whole mount immunostaining of PECAM1 in the embryos and yolk sac fixed in 4% PFA at 4 °C overnight was carried out as described earlier^65^. The vasculature in retinal flat mounts was visualized by IB4 staining. Tip cells were detected using antibodies against the ESM1 marker protein (Supplementary Table 2). For quantification of sprouting angiogenesis in the retina, digital images of the flat mounts were evaluated using Adobe Photoshop. Areas of normal vessel growth and hypersprouting were selected using the lasso and quick selection tools. Size of these areas measured in pixels was obtained from the histogram window. Quantitative analysis of the vascular network regarding vascular end points was performed in the selected areas using the free software AngioTool (National Institute of Health National Cancer Institute, Gaithersburg, MD).

### Whole mount in situ hybridization

For WISH, embryos were processed according to the protocol provided in the MAMEP database (http://mamep.molgen.mpg.de). The probe for *Wwc2* corresponds to nucleotides 2085-3230 of NM_133791.4. The probe template was produced by PCR with a reverse primer containing a T7 site for antisense transcription. A DIG-labeled probe was generated by in vitro transcription according to standard procedures, and staining was performed using BM Purple (Roche). Following the staining reaction, samples were postfixed and stored in 4% PFA/PBS. For imaging of WISH-stained embryos, a MZ16A dissection microscope (Leica) fitted with an AxioCam MRc5 (Carl Zeiss MicroImaging) was used in combination with the AxioVision Software (Carl Zeiss MicroImaging) for image processing.

### RNA isolation and quantitative RT-PCR reaction

Total RNA was isolated from embryonic or placental tissue using the GenElute Mammalian total RNA Miniprep Kit according to the manufacturer’s instructions. cDNA was synthesized using the Superscript III Reverse Transcriptase kit (Invitrogen). SYBR Green (Applied Biosystems) dye-based qRT-PCR was performed by the core facility genomics (University Hospital Münster, Germany) using CFX384 Touch (Biorad). The used primers are listed in Table 1 of the Supporting Information. Each cDNA sample was measured in triplicates and data are expressed relative to the housekeeping gene GAPDH. The samples from control and *Wwc2* KO mice were compared using the comparative C_T_ method (2^−ΔΔC^_T_ = [(C_T_ gene of interest – C_T_ internal control) sample – (C_T_ gene of interest – C_T_ internal control) sample B).

### Transcriptome analysis

In order to determine the changes in the transcriptome after *Wwc2* ablation, RNA from three wildtype, three heterozygous, and four homozygous *Wwc2* KO embryos at E11.5 was isolated and analyzed by RNAseq analysis. Library preparation of the total RNA was performed with the NEBNext Ultra RNA directional Kit and paired read sequencing was performed using a NextSeq^®^ 500 System with a read length of 1 × 80 bp. Using a molecular barcode, the samples were demultiplexed (bcl2fastq2) to fastq data and quality controlled (FastQC). Trimmomatic was used for adapter trimming and read filtering^66^. The resulting reads were aligned to the mouse reference genome (mm10) using Hisat2^67^ and the aligned reads were sorted using samtools^68^. The sorted and aligned reads were counted into genes using htsec-counts^69^. The test for differential expression were performed using the r-package deseq2^70^.

### Tissue extract preparation and western blot analysis

Tissue extract preparation from embryos was performed as described earlier^14^. In brief, tissues were lysed in ice-cold lysis buffer (20 mM Tris-HCl pH 7.4, 20 mM NaCl, 1 mM EDTA, 50 mM NaF, 10 mM Na_4_P_2_O_7_, 1% Triton-X-100) containing protease (Roche) and phosphatase (Sigma) inhibitors. After the lysates were centrifuged at 14,000 × *g* for 45 min, supernatants were removed and stored at −80 °C until further use. Western Blot analysis was performed using standard protocols. Antibodies used are listed in Supplementary Tab. 2.

### Statistical analysis

For statistical analysis, the software GraphPad Prism 5 was used to perform a paired two-tailed Student’s *t* test. Data describing vascular networks were evaluated using GraphPad Prism 7 (Kruskal–Wallis test and Dunn’s multiple comparisons test) and AngioTool^71^.

## Supplementary information

Combined supplementary information

## Supplementary information

**Supplementary information** The online version contains supplementary material available at 10.1038/s41419-021-03409-0.

## References

[1] Cockburn K, Rossant J (2010). Making the blastocyst: lessons from the mouse. J. Clin. Invest..

[2] Takaoka K, Hamada H (2012). Cell fate decisions and axis determination in the early mouse embryo. Development.

[3] Nishioka N (2009). The Hippo signaling pathway components Lats and Yap pattern Tead4 activity to distinguish mouse trophectoderm from inner cell mass. Dev. Cell.

[4] Wicklow E (2014). Hippo pathway members restrict SOX2 to the inner cell mass where it promotes ICM fates in the mouse blastocyst. PLoS Genet..

[5] Posfai E (2017). Position- and Hippo signaling-dependent plasticity during lineage segregation in the early mouse embryo. Elife.

[6] Sasaki H (2017). Roles and regulations of Hippo signaling during preimplantation mouse development. Dev. Growth Differ..

[7] Irvine KD, Harvey KF (2015). Control of organ growth by patterning and hippo signaling in Drosophila. Cold Spring Harb. Perspect. Biol..

[8] Misra JR, Irvine KD (2018). The Hippo signaling network and its biological functions. Annu. Rev. Genet..

[9] Fu V, Plouffe SW, Guan KL (2017). The Hippo pathway in organ development, homeostasis, and regeneration. Curr. Opin. Cell Biol..

[10] Ma S, Meng Z, Chen R, Guan KL (2019). The Hippo pathway: biology and pathophysiology. Annu. Rev. Biochem..

[11] Meng Z, Moroishi T, Guan KL (2016). Mechanisms of Hippo pathway regulation. Genes Dev..

[12] Bae SJ, Luo X (2018). Activation mechanisms of the Hippo kinase signaling cascade. Biosci. Rep..

[13] Wennmann DO (2014). Evolutionary and molecular facts link the WWC protein family to Hippo signaling. Mol. Biol. Evol..

[14] Hermann A (2018). WW and C2 domain-containing proteins regulate hepatic cell differentiation and tumorigenesis through the hippo signaling pathway. Hepatology.

[15] Zhang L (2014). KIBRA: In the brain and beyond. Cell Signal..

[16] Makuch L (2011). Regulation of AMPA receptor function by the human memory-associated gene KIBRA. Neuron.

[17] Vogt-Eisele A (2014). KIBRA (KIdney/BRAin protein) regulates learning and memory and stabilizes Protein kinase Mζ. J. Neurochem.

[18] Cao J (2019). The single-cell transcriptional landscape of mammalian organogenesis. Nature.

[19] Lallemand Y, Luria V, Haffner-Krausz R, Lonai P (1998). Maternally expressed PGK-Cre transgene as a tool for early and uniform activation of the Cre site-specific recombinase. Transgenic Res..

[20] Lan ZJ, Xu X, Cooney AJ (2004). Differential oocyte-specific expression of Cre recombinase activity in GDF-9-iCre, Zp3cre, and Msx2Cre transgenic mice. Biol. Reprod..

[21] Perez-Garcia V (2018). Placentation defects are highly prevalent in embryonic lethal mouse mutants. Nature.

[22] Papaioannou VE, Behringer RR (2012). Early embryonic lethality in genetically engineered mice: diagnosis and phenotypic analysis. Vet. Pathol..

[23] Rossant J, Cross JC (2001). Placental development: lessons from mouse mutants. Nat. Rev. Genet..

[24] Ward JM, Elmore SA, Foley JF (2012). Pathology methods for the evaluation of embryonic and perinatal developmental defects and lethality in genetically engineered mice. Vet. Pathol..

[25] Maltepe E, Bakardjiev AI, Fisher SJ (2010). The placenta: transcriptional, epigenetic, and physiological integration during development. J. Clin. Invest..

[26] Woods L, Perez-Garcia V, Hemberger M (2018). Regulation of placental development and its impact on fetal growth-new insights from mouse models. Front. Endocrinol..

[27] Rossant J, Hirashima M (2003). Vascular development and patterning: making the right choices. Curr. Opin. Genet. Dev..

[28] Hemberger M, Hanna CW, Dean W (2020). Mechanisms of early placental development in mouse and humans. Nat. Rev. Genet..

[29] Walentin K (2015). A Grhl2-dependent gene network controls trophoblast branching morphogenesis. Development.

[30] Tai-Nagara I (2017). Placental labyrinth formation in mice requires endothelial FLRT2/UNC5B signaling. Development.

[31] Azevedo Portilho N, Pelajo-Machado M (2018). Mechanism of hematopoiesis and vasculogenesis in mouse placenta. Placenta.

[32] Gertsenstein M (2015). Mouse embryos’ fusion for the tetraploid complementation assay. Methods Mol. Biol..

[33] Gheorghe C, Mohan S, Longo LD (2006). Gene expression patterns in the developing murine placenta. J. Soc. Gynecol. Investig..

[34] Soncin F (2018). Comparative analysis of mouse and human placentae across gestation reveals species-specific regulators of placental development. Development.

[35] Sörensen I, Adams RH, Gossler A (2009). DLL1-mediated Notch activation regulates endothelial identity in mouse fetal arteries. Blood.

[36] Ribatti D, Crivellato E (2012). “Sprouting angiogenesis”, a reappraisal. Dev. Biol..

[37] Chen W (2019). The endothelial tip-stalk cell selection and shuffling during angiogenesis. J. Cell Commun. Signal.

[38] Gerhardt H (2003). VEGF guides angiogenic sprouting utilizing endothelial tip cell filopodia. J. Cell Biol..

[39] Jakobsson L (2010). Endothelial cells dynamically compete for the tip cell position during angiogenic sprouting. Nat. Cell Biol..

[40] Kim J (2017). YAP/TAZ regulates sprouting angiogenesis and vascular barrier maturation. J. Clin. Invest..

[41] Rocha SF (2014). Esm1 modulates endothelial tip cell behavior and vascular permeability by enhancing VEGF bioavailability. Circ. Res..

[42] McPherson JP (2004). Lats2/Kpm is required for embryonic development, proliferation control and genomic integrity. EMBO J..

[43] Morin-Kensicki EM (2006). Defects in yolk sac vasculogenesis, chorioallantoic fusion, and embryonic axis elongation in mice with targeted disruption of Yap65. Mol. Cell Biol..

[44] Nishio M (2012). Cancer susceptibility and embryonic lethality in Mob1a/1b double-mutant mice. J. Clin. Invest..

[45] Lee JH (2008). A crucial role of WW45 in developing epithelial tissues in the mouse. EMBO J..

[46] Oh S (2009). Crucial role for Mst1 and Mst2 kinases in early embryonic development of the mouse. Mol. Cell Biol..

[47] Park JA, Kwon YG (2018). Hippo-YAP/TAZ signaling in angiogenesis. BMB Rep..

[48] Azad T, Ghahremani M, Yang X (2019). The Role of YAP and TAZ in angiogenesis and vascular mimicry. Cells.

[49] Boopathy GTK, Hong W (2019). Role of Hippo pathway-YAP/TAZ signaling in angiogenesis. Front. Cell Dev. Biol..

[50] Wang X (2017). YAP/TAZ orchestrate VEGF signaling during developmental angiogenesis. Dev. Cell.

[51] Sakabe M (2017). YAP/TAZ-CDC42 signaling regulates vascular tip cell migration. Proc. Natl Acad. Sci. USA.

[52] Chazaud C, Yamanaka Y (2016). Lineage specification in the mouse preimplantation embryo. Development.

[53] Bratt A (2005). Angiomotin regulates endothelial cell-cell junctions and cell motility. J. Biol. Chem..

[54] Hirate Y (2013). Polarity-dependent distribution of angiomotin localizes Hippo signaling in preimplantation embryos. Curr. Biol..

[55] Moleirinho S (2017). Regulation of localization and function of the transcriptional co-activator YAP by angiomotin. Elife.

[56] Mana-Capelli S, Paramasivam M, Dutta S, McCollum D (2014). Angiomotins link F-actin architecture to Hippo pathway signaling. Mol. Biol. Cell.

[57] Blanco R, Gerhardt HVEGF (2013). and Notch in tip and stalk cell selection. Cold Spring Harb. Perspect. Med..

[58] Betz C, Lenard A, Belting HG, Affolter M (2016). Cell behaviors and dynamics during angiogenesis. Development.

[59] Sewduth R, Santoro MM (2016). “Decoding” angiogenesis: new facets controlling endothelial cell behavior. Front. Physiol..

[60] Park MH (2019). CCN1 interlinks integrin and hippo pathway to autoregulate tip cell activity. Elife.

[61] Virnicchi G, Bora P, Gahurova L, Šušor A, Bruce AW (2020). Wwc2 is a novel cell division regulator during preimplantation mouse embryo lineage formation and oogenesis. Front. Cell Dev. Biol..

[62] Summers MC, McGinnis LK, Lawitts JA, Raffin M, Biggers JD (2000). IVF of mouse ovar in a simplex optimized medium supplemented with amino acids. Hum. Reprod..

[63] Nagy A, Rossant J, Nagy R, Abramow-Newerly W, Roder JC (1993). Derivation of completely cell culture-derived mice from early-passage embryonic stem cells. Proc. Natl Acad. Sci. USA.

[64] Pitulescu ME, Schmidt I, Benedito R, Adams RH (2010). Inducible gene targeting in the neonatal vasculature and analysis of retinal angiogenesis in mice. Nat. Protoc..

[65] Wu ZQ (2014). A Snail1/Notch1 signalling axis controls embryonic vascular development. Nat. Commun..

[66] Bolger, A. M., Lohse, M., & Usadel, B. Trimmomatic: a flexible trimmer for Illumina Sequence Data. Bioinformatics 2014;btu170. 10.1093/bioinformatics/btu170 PMC4103590 24695404

[67] Langmead B, Salzberg S (2012). Fast gapped-read alignment with Bowtie 2. Nat. Methods.

[68] Li H (2011). A statistical framework for SNP calling, mutation discovery, association mapping and population genetical parameter estimation from sequencing data. Bioinformatics.

[69] Anders S, Pyl TP, Huber W (2015). HTSeq-A Python framework to work with high-throughput sequencing data. Bioinformatics.

[70] Love MI, Huber W, Anders S (2014). Moderated estimation of fold change and dispersion for RNA-seq data with DESeq2. Genome Biol..

[71] Zudaire E., Gambardella L., Kurcz C., Vermeren S. A computational tool for quantitative analysis of vascular networks. *PLoS ONE* 2011;6e27385 10.1371/journal.pone.0027385 PMC3217985 22110636

