# Supplementary material for: The Hippo pathway component Wwc2 is a key regulator of embryonic development and angiogenesis in mice
Source: Cell Death Dis. 2021 Jan 22;12(1):117. doi: 10.1038/s41419-021-03409-0 (PMC7822818; doi:10.1038/s41419-021-03409-0)
Supplement: Supplementary file 1 — Combined supplementary information [file 41419_2021_3409_MOESM1_ESM.docx]

**SUPPLEMENTARY INFORMATION**

### Supplementary Table 1: Primer used for genotyping and qPCR-RT analysis

**Genoyting**

| Gene | Sequence |
| --- | --- |
| WWC1 geno 1/2 fwd | TCAGTGCTAGTGAACTGCTGC |
| WWC1 geno 1 rev | CTCACAGGAGGGGCTTGGATGC |
| WWC1 geno 2 rev | GGATGGCTACACGGATGTTCC |
| WWC2 29 fwd | TGCTTGGCTGGAGCTG |
| WWC2 33 fwd | AGTTCTCTCGGTCTCG |
| WWC2 34 rev | CAAACCAGAACTGAGC |
| WWC2F31 | ACTGCTTGGCTGTGTTAGAGC |
| WWC2F88 | CAGTGGGGCTGTGTCTATGG |
| WWC2R1074 | ATCCTCAGGCAGACCTAAACC |
| Cre PCR fwd | GACCAGGTTCGTTCACTCA |
| Cre PCR rev | TAGCGCCGTAAATCAA |

**qRT-PCR**

| Gene | Forward Primer | Reverse Primer |
| --- | --- | --- |
| Birc5 | GAACCCGATGACAACCCGAT | TGGTCTCCTTTGCAATTTTGTTC |
| Ctgf | CCTAGCTGCCTACCGACTG | CAAACTTGACAGGCTTGGCG |
| Cyr61 | CACTGAAGAGGCTTCCTGTCTT | GATCCGGGTCTCTTTCACCA |
| Gapdh | TGGCCTTCCGTGTTCCTACC | GGTCCTCAGTGTAGCCCAAGATG |
| Flt1 | GAGGAGGATGAGGGTGTCTATAGGT | GTGATCAGCTCCAGGTTTGACTT |
| Junb | AGGTGAAGACACTCAAGGCTGAGAA | TGACATGGGTCATGACCTTCTGCTT |
| Krt19 | GGGGGTTCAGTACGCATTGG | GAGGACGAGGTCACGAAGC |
| Pecam1 | AGGCTTGCATAGAGCTCCAG | TTCTTGGTTTCCAGCTATGG |
| Vegfa | CGATTGAGACCCTGGTGGAC | ATCCGCATGATCTGCATGGT |
| Wwc2 | CCAGGACGCACTCAGGAC | GCTAGATGAAGACCCTGAGAAGA |

### Supplementary Table 2: Antibodies and antibody conjugates used in this study

**Primary antibodies**

| Antigen | Supplier/Reference | Host |
| --- | --- | --- |
| pan-cytokeratin (CK) | Santa Cruz Biotechnology, Inc., Heidelberg, Germany | mouse |
| PECAM1 | BD Biosciences, CA, USA | rat |
| SMA | Sigma-Aldrich Chemie GmbH, Munich, Germany | mouse |
| WWC2 | Hermann et al., 2019 | rabbit |

### Secondary antibodies

| Antibody conjugates and Antigen | Supplier |
| --- | --- |
| Alexa Fluor® 488 - α-Mouse IgG | Invitrogen, Darmstadt, Germany |
| Alexa Fluor® 488 - α-Rabbit IgG | Invitrogen, Darmstadt, Germany |
| Alexa Fluor® 488 - α-Rat IgG | Invitrogen, Darmstadt, Germany |
| Alexa Fluor® 594 - α-Mouse IgG | Invitrogen, Darmstadt, Germany |
| Alexa Fluor® 594 - α-Rabbit IgG | Invitrogen, Darmstadt, Germany |
| Alexa Fluor® 594 - α-Rat IgG | Invitrogen, Darmstadt, Germany |
| HRP - α-Mouse IgG | Jackson Immunoresearch Laboratories, Inc., Suffolk, GB |
| HRP - α-Rabbit IgG | Jackson Immunoresearch Laboratories, Inc., Suffolk, GB |
| Biotin-conjugated rabbit anti-rat IgG | Dianova GmbH, Hamburg, Germany |

| Conjugates | Supplier |
| --- | --- |
| IB4 (biotinylated) | Vector Laboratories, Burligame, USA |
| Streptavidin-Cy3 | Sigma-Aldrich Chemie GmbH, Munich, Germany |

**Supplementary Table 3: Genotype analysis of embryos at different stages of development.**

|  | **Total** | **+/+** | **+/-** | **-/-** |
| --- | --- | --- | --- | --- |
| **E13.5** | 25 | 10 | 14 | 1 |
| **E12.5** | 17 | 6 | 10 | 1 |
| **E11.5** | 221 | 34 | 134 | 53 |
| **E10.5** | 16 | 2 | 9 | 5 |
| **E9.5** | 75 | 11 | 48 | 16 |
| **E8.5** | 65 | 3 | 41 | 21 |
| **E6.5** | 45 | 1 | 18 | 26 |

E, Embryonic day; +/+, WWC2 wildtype (= Zp3-cre-WWC2^+/+^); -/+, heterozygous WWC2 KO (Zp3-cre-WWC2^fl/+^); -/-, homozygous WWC2 KO (= Zp3-cre-WWC2^fl/fl^).

**Supplementary Table 4**: **Results from the tetraploid complementation assay.**

Es cells with heterozygous (+/-) or homozygous (-/-) WWC2 KO were used for the aggregation assay and blastocysts were transferred into the uterus of pseudopregnant mice. No E19.5 embryos were found when WWC2 -/- Es cells were used.

| ES cell line | Aggregated cells | Tranferred blastocytes | E19.5 embryos |
| --- | --- | --- | --- |
| B3 (WWC2 -/+) | 43 | 43 | 16 |
| A1 (WWC2-/-) | 44 | 44 | 0 |
| C3 (WWC2-/-) | 27 | 27 | 0 |
| D3 (WWC2-/-) | 44 | 44 | 0 |

**Supplementary Table 5:** List of the 30 most upregulated genes in WWC2 KO embryos (E11.5) with FDR corrected p-value < 0.05. Genes normaly expressed in the placenta are marked in red.

| **#** | **Gene** | **Log2(Fold change)** | **Full name** |
| --- | --- | --- | --- |
| 1 | **Enpp7** | 7,597 | ectonucleotide pyrophosphatase/phosphodiesterase 7 |
| 2 | **Psg18** | 7,583 | pregnancy specific glycoprotein 18 |
| 3 | **Hspa1a** | 7,488 | heat shock protein 1A |
| 4 | **Prl7c1** | 7,452 | prolactin family 7, subfamily c, member 1 |
| 5 | **Psg21** | 7,357 | pregnancy-specific glycoprotein 21 |
| 6 | **Psg27** | 7,185 | pregnancy-specific glycoprotein 27 |
| 7 | **Ceacam12** | 7,019 | carcinoembryonic antigen-related cell adhesion molecule 12 |
| 8 | **Psg16** | 6,966 | pregnancy specific glycoprotein 16 |
| 9 | **Tpbpb** | 6,906 | trophoblast specific protein beta |
| 10 | **Psg23** | 6,881 | pregnancy-specific glycoprotein 23 |
| 11 | **Psg-ps1** | 6,819 | pregnancy specific glycoprotein, pseudogene 1 |
| 12 | **Mmp1a** | 6,742 | matrix metallopeptidase 1a (interstitial collagenase) |
| 13 | **Ceacam14** | 6,724 | carcinoembryonic antigen-related cell adhesion molecule 14 |
| 14 | **Prl8a8** | 6,610 | prolactin family 8, subfamily a, member 81 |
| 15 | **Psg17** | 6,542 | pregnancy specific glycoprotein 17 |
| 16 | **Ido1** | 6,526 | indoleamine 2,3-dioxygenase 1 |
| 17 | **Prl7b1** | 6,521 | prolactin family 7, subfamily b, member 1 |
| 18 | **Psg25** | 6,493 | pregnancy-specific glycoprotein 25 |
| 19 | **Cts3** | 6,488 | cathepsin 3 |
| 20 | **Gm3383** | 6,457 | predicted gene 3383 |
| 21 | **Ceacam13** | 6,453 | carcinoembryonic antigen-related cell adhesion molecule 13 |
| 22 | **Prl7a2** | 6,436 | prolactin family 7, subfamily a, member 2 |
| 23 | **Ccr1l1** | 6,419 | chemokine (C-C motif) receptor 1-like 1 |
| 24 | **Ceacam11** | 6,415 | carcinoembryonic antigen-related cell adhesion molecule 11 |
| 25 | **Psg28** | 6,405 | pregnancy-specific glycoprotein 28 |
| 26 | **Ctsm** | 6,377 | cathepsin M |
| 27 | **Ube2d2b** | 6,333 | ubiquitin-conjugating enzyme E2D 2B |
| 28 | **Tpbpa** | 6,326 | trophoblast specific protein alpha |
| 29 | **Cyp24a1** | 6,287 | cytochrome P450, family 24, subfamily a, polypeptide 1 |
| 30 | **Ceacam5** | 6,226 | carcinoembryonic antigen-related cell adhesion molecule 5 |

**Supplementary Table 6:** List of the 30 most downregulated genes in WWC2 KO embryos (E11.5) with FDR corrected p-value < 0.05. Genes with a function in the nervous system are marked in red.

| **#** | **Gene** | **Log2(Fold change)** | | **Full name** |
| --- | --- | --- | --- | --- |
| 1 | **Neurod6** | -4,574 | | neurogenic differentiation 6 |
| 2 | **Enam** | -4,376 | | enamelin |
| 3 | **Tm4sf4** | -3,636 | | transmembrane 4 superfamily member 4 |
| 4 | **Cyp2d10** | -3,478 | | cytochrome P450, family 2, subfamily d, polypeptide 10 |
| 5 | **Agxt** | -3,464 | | alanine-glyoxylate aminotransferase |
| 6 | **C8b** | -3,333 | | complement component 8, beta polypeptide |
| 7 | **Hbq1a** | -3,237 | | hemoglobin, theta 1A |
| 8 | **Crybb1** | -3,220 | | crystallin, beta B1 |
| 9 | **Crybb3** | -3,190 | | crystallin, beta B3 |
| 10 | **Kcns2** | -3,136 | | K+ voltage-gated channel, subfamily S, 2 |
| 11 | **Crh** | -3,099 | | corticotropin releasing hormone |
| 12 | **F830016B08Rik** | | -3,060 | RIKEN cDNA F830016B08 gene |
| 13 | **Cryba1** | -2,917 | | crystallin, beta A1 |
| 14 | **Kera** | -2,914 | | keratocan |
| 15 | **Npy** | -2,892 | | neuropeptide Y |
| 16 | **Pcdha9** | -2,875 | | protocadherin alpha 9 |
| 17 | **Gja8** | -2,870 | | gap junction protein, alpha 8 |
| 18 | **Timd4** | -2,852 | | T cell immunoglobulin and mucin domain containing 4 |
| 19 | **Serpina6** | -2,848 | | serine (or cysteine) peptidase inhibitor, clade A, member 6 |
| 20 | **9830132P13Rik** | -2,808 | | RIKEN cDNA 9830132P13 gene |
| 21 | **Pnlip** | -2,748 | | pancreatic lipase |
| 22 | **Matn4** | -2,746 | | matrilin 4 |
| 23 | **C8a** | -2,719 | | complement component 8, alpha polypeptide |
| 24 | **Alb** | -2,716 | | albumin |
| 25 | **Neurod2** | -2,695 | | neurogenic differentiation 2 |
| 26 | **Matn1** | -2,645 | | matrilin 1, cartilage matrix protein |
| 27 | **Ugt2b35** | -2,605 | | UDP glucuronosyltransferase 2 family, polypeptide B35 |
| 28 | **Scn10a** | -2,591 | | sodium channel, voltage-gated, type X, alpha |
| 29 | **Mir483** | -2,575 | | microRNA 483 |
| 30 | **Ngp** | -2,568 | | neutrophilic granule protein |

**Supplementary Table 7: Positive enriched gene sets identified using GSEA.**

The GSEA of the DEG against 6157 gene sets of Gene Ontology: Biological Process used in this analysis resulted in 504 positive and 252 negative enriched genes sets significant at FDR < 25% (False Discovery Rate). The top 30 enriched gene sets with the highest NES (normalized enrichment score) are shown. Results were compared to the pervious analysis with the DAVID annotation Tool. Identical gene sets are marked in red, gene sets leading to a similar conclusion are marked in yellow.

|  | **Gene Set** | **NES** | **FDR** |
| --- | --- | --- | --- |
| 1 | GO_PLACENTA_DEVELOPMENT | 2.73 | 0.000 |
| 2 | GO_MULTI_MULTICELLULAR_ORGANISM_PROCESS | 2.69 | 0.000 |
| 3 | GO_RESPONSE_TO_BACTERIUM | 2.45 | 0.005 |
| 4 | GO_EMBRYONIC_PLACENTA_DEVELOPMENT | 2.43 | 0.005 |
| 5 | GO_CYTOKINE_MEDIATED_SIGNALING_PATHWAY | 2.41 | 0.005 |
| 6 | GO_REGULATION_OF_GENE_SILENCING | 2.38 | 0.007 |
| 7 | GO_EMBRYO_IMPLANTATION | 2.35 | 0.010 |
| 8 | GO_INFLAMMATORY_RESPONSE | 2.35 | 0.009 |
| 9 | GO_RESPONSE_TO_BIOTIC_STIMULUS | 2.34 | 0.008 |
| 10 | GO_REPRODUCTIVE_SYSTEM_DEVELOPMENT | 2.33 | 0.008 |
| 11 | GO_CELL_DIFFERENTIATION_INVOLVED_IN_EMBRYONIC_PLACENTA_DEVELOPMENT | 2.32 | 0.008 |
| 12 | GO_TOLERANCE_INDUCTION | 2.28 | 0.013 |
| 13 | GO_DEFENSE_RESPONSE | 2.27 | 0.014 |
| 14 | GO_RESPONSE_TO_MOLECULE_OF_BACTERIAL_ORIGIN | 2.26 | 0.014 |
| 15 | GO_MULTI_ORGANISM_PROCESS | 2.25 | 0.015 |
| 16 | GO_POSITIVE_REGULATION_OF_LEUKOCYTE_CHEMOTAXIS | 2.22 | 0.020 |
| 17 | GO_LIPID_CATABOLIC_PROCESS | 2.22 | 0.021 |
| 18 | GO_NEGATIVE_REGULATION_OF_DNA_BINDING_TRANSCRIPTION_FACTOR_ACTIVITY | 2.21 | 0.022 |
| 19 | GO_REPRODUCTION | 2.20 | 0.022 |
| 20 | GO_CELLULAR_RESPONSE_TO_OXYGEN_LEVELS | 2.20 | 0.022 |
| 21 | GO_ANTERIOR_POSTERIOR_PATTERN_SPECIFICATION | 2.19 | 0.023 |
| 22 | GO_REGULATION_OF_CELL_CELL_ADHESION | 2.17 | 0.027 |
| 23 | GO_RESPONSE_TO_EXTRACELLULAR_STIMULUS | 2.16 | 0.027 |
| 24 | GO_POSITIVE_REGULATION_OF_INTERLEUKIN_8_PRODUCTION | 2.16 | 0.026 |
| 25 | GO_GENE_SILENCING | 2.16 | 0.026 |
| 26 | GO_RESPONSE_TO_OXYGEN_LEVELS | 2.16 | 0.026 |
| 27 | GO_OXIDATION_REDUCTION_PROCESS | 2.14 | 0.031 |
| 28 | GO_BLOOD_VESSEL_MORPHOGENESIS | 2.13 | 0.031 |
| 29 | GO_DEFENSE_RESPONSE_TO_OTHER_ORGANISM | 2.12 | 0.034 |
| 30 | GO_VASCULATURE_DEVELOPMENT | 2.12 | 0.034 |

**Supplementary Table 8: Negative enriched gene sets identified using GSEA.**

The top 30 negativly enriched gene sets with the highest NES (normalized enrichment score) are shown. Results were compared to the pervious analysis with the DAVID annotation Tool. Identical gene sets are marked in red, gene sets leading to a similar conclusion are marked in yellow. (FDR=False Discovery Rate).

|  | GS | NES | FDR |
| --- | --- | --- | --- |
| 1 | GO_SYNAPTIC_SIGNALING | -4.56 | 0.000 |
| 2 | GO_SYNAPSE_ORGANIZATION | -4.10 | 0.000 |
| 3 | GO_SYNAPSE_ASSEMBLY | -4.07 | 0.000 |
| 4 | GO_REGULATION_OF_ION_TRANSMEMBRANE_TRANSPORT | -3.76 | 0.000 |
| 5 | GO_NEURON_DIFFERENTIATION | -3.49 | 0.000 |
| 6 | GO_GLUTAMATE_RECEPTOR_SIGNALING_PATHWAY | -3.48 | 0.000 |
| 7 | GO_REGULATION_OF_SYNAPSE_STRUCTURE_OR_ACTIVITY | -3.45 | 0.000 |
| 8 | GO_PROTEIN_LOCALIZATION_TO_SYNAPSE | -3.36 | 0.000 |
| 9 | GO_REGULATION_OF_TRANS_SYNAPTIC_SIGNALING | -3.34 | 0.000 |
| 10 | GO_REGULATION_OF_NERVOUS_SYSTEM_DEVELOPMENT | -3.33 | 0.000 |
| 11 | GO_REGULATION_OF_NEURON_DIFFERENTIATION | -3.29 | 0.000 |
| 12 | GO_POSITIVE_REGULATION_OF_NERVOUS_SYSTEM_DEVELOPMENT | -3.29 | 0.000 |
| 13 | GO_REGULATION_OF_NEUROTRANSMITTER_RECEPTOR_ACTIVITY | -3.26 | 0.000 |
| 14 | GO_PROTEIN_TARGETING_TO_MEMBRANE | -3.26 | 0.000 |
| 15 | GO_NEUROGENESIS | -3.25 | 0.000 |
| 16 | GO_DENDRITE_DEVELOPMENT | -3.25 | 0.000 |
| 17 | GO_ADULT_BEHAVIOR | -3.25 | 0.000 |
| 18 | GO_REGULATION_OF_SYNAPSE_ASSEMBLY | -3.16 | 0.000 |
| 19 | GO_RECEPTOR_LOCALIZATION_TO_SYNAPSE | -3.15 | 0.000 |
| 20 | GO_NEURON_DEVELOPMENT | -3.14 | 0.000 |
| 21 | GO_REGULATION_OF_TRANSMEMBRANE_TRANSPORT | -3.14 | 0.000 |
| 22 | GO_VESICLE_MEDIATED_TRANSPORT_IN_SYNAPSE | -3.13 | 0.000 |
| 23 | GO_REGULATION_OF_SYNAPTIC_VESICLE_CYCLE | -3.12 | 0.000 |
| 24 | GO_REGULATION_OF_GLUTAMATE_RECEPTOR_SIGNALING_PATHWAY | -3.03 | 0.000 |
| 25 | GO_NERVOUS_SYSTEM_PROCESS | -3.02 | 0.000 |
| 26 | GO_PRESYNAPSE_ORGANIZATION | -3.01 | 0.000 |
| 27 | GO_ESTABLISHMENT_OF_PROTEIN_LOCALIZATION_TO_ENDOPLASMIC_RETICULUM | -3.00 | 0.000 |
| 28 | GO_PROTEIN_LOCALIZATION_TO_ENDOPLASMIC_RETICULUM | -3.00 | 0.000 |
| 29 | GO_NEUROTRANSMITTER_SECRETION | -2.98 | 0.000 |
| 30 | GO_COTRANSLATIONAL_PROTEIN_TARGETING_TO_MEMBRANE | -2.98 | 0.000 |


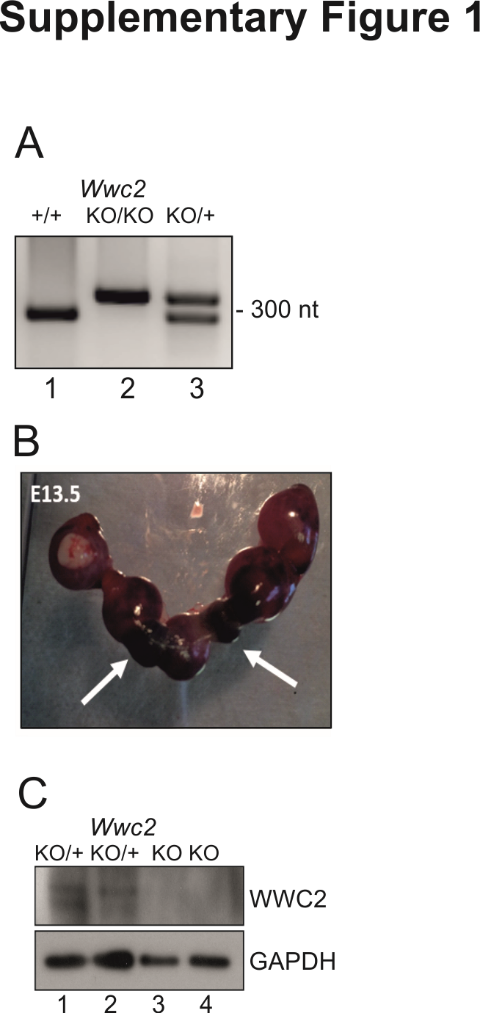


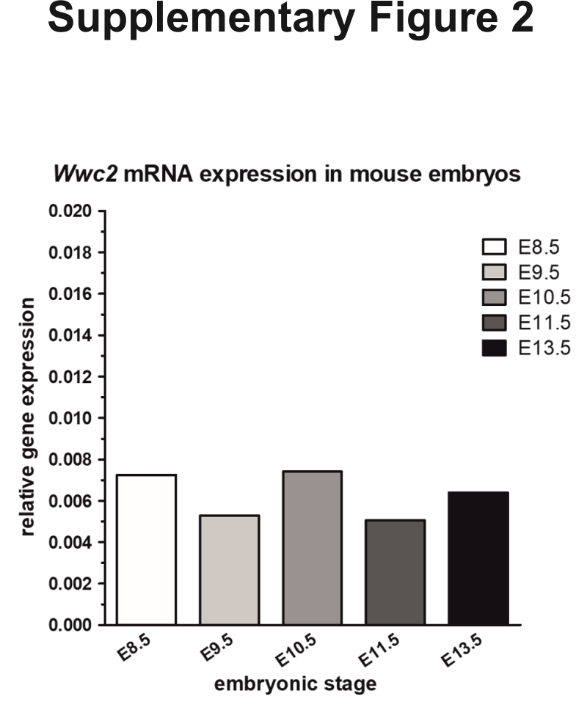


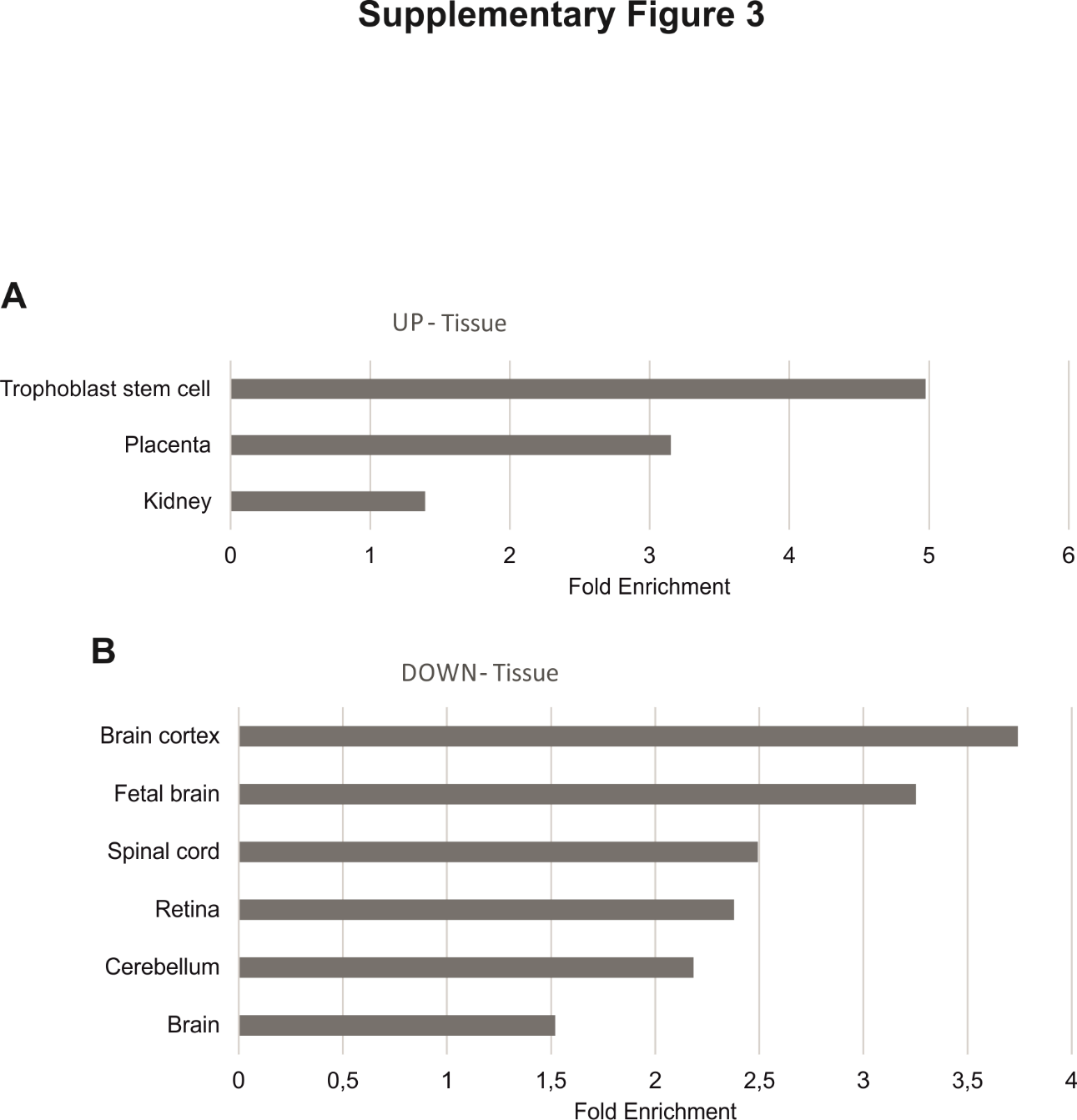


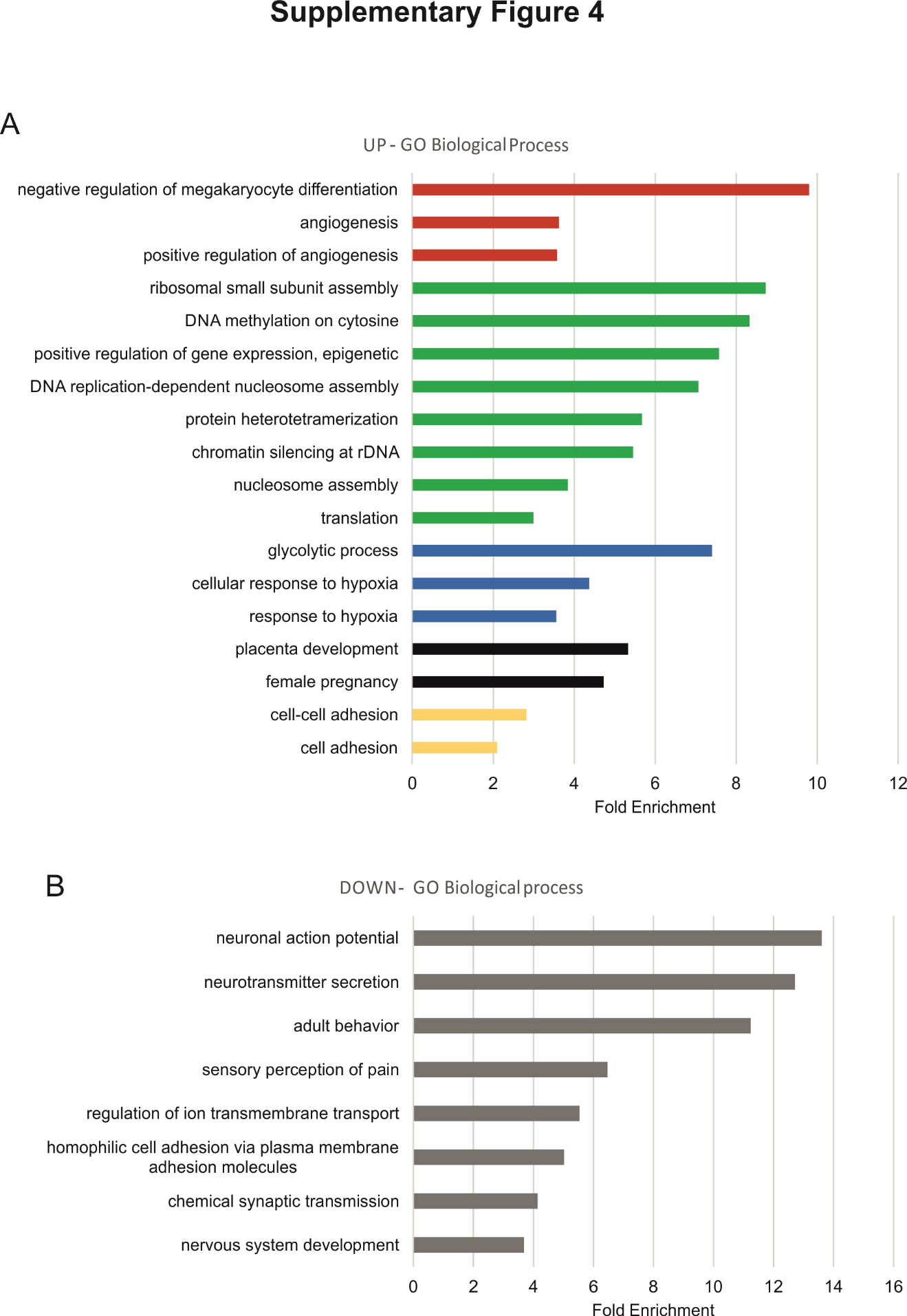


**Supplementary Figure Legends**

**Supplementary Figure S1: Characterization of the Wwc2 KO phenotype in dissected embryos**

**A)** Genotyping of genomic DNA from E11.5 embryos. PCR products corresponding to samples from Wwc2 wildtype (+/+: 267 bp, lane 1), homozygous Wwc2 KO (KO/KO: 325 bp, lane 2) and Wwc2 heterozygous KO embryos (KO/+: 267 bp and 325 bp, lane 3) are indicated. **B)** Exemplary presentation of an isolated mouse uterus with embryos at E13.5. Arrows indicate the presence of degenerated Wwc2 KO embryos. **C)** Western blot analysis with samples from E11.5 embryos indicates the lack of WWC2 protein in the Wwc2 KO lysates (lanes 3 and 4). GAPDH detection served as an internal loading control.

**Supplementary Figure S2: *Wwc2* mRNA expression during mouse embryogenesis**

Whole mRNA was isolated from wild type mouse embryos at embryonal stages E8.5 to E13.5 and was used for qRT-PCR analysis on *Wwc2* expression. For this approach, three embryos from the specific embryonic stages were pooled and isolated mRNA were used for qRT-PCR.

**Supplementary Figure 3: Tissue-specific gene set enrichment analysis of the up- and downregulated genes in Wwc2-deficient embryos (E11.5)**

For the analysis, genes with an FDR-corrected p-value < 0.05 and a log2(FC) > 1 were taken into account (1984 genes). Fold enrichment scores of all terms from Uniprot category “Tissue” with FDR corrected p-values < 0.001 are shown in the graph. **A)** Analysis of upregulated genes (UP). **B)** Analysis of downregulated genes (DOWN). Analysis was performed using the DAVID Bioinformatics Rescources.

**Supplementary Figure 4: Gene set enrichment analysis of the GO category “Biological Process” of the up- and downregulated genes in Wwc2 KO embryos (E11.5)**

For the analysis, the genes with an FDR corrected p-value < 0.05 and a log2(FC) > 1 were taken into account (1984 genes). Fold enrichment of all terms from GO category “Biological Process” with FDR-corrected p-values <0.001 are shown in the bar graph. **A)** Analysis of upregulated genes (UP). Terms can be divided into five groups: angiogenesis (marked in red), transcription and translation (marked in green), metabolism (marked in blue), placenta development (marked in black) and cell adhesion (marked in yellow). **B)** Analysis of downregulated genes (DOWN). Analyses were performed using the DAVID Bioinformatics Rescources.
